# Diet and Nutrition: Olestra’s Second Wind

**Published:** 2005-08

**Authors:** Carol Potera

Olestra, the nonabsorbable fat substitute, has had a rocky past. Originally explored as a cholesterol-lowering drug, olestra was approved in 1996 for use in fat-free snack foods with the proviso that these snacks carry a warning about possible cramping and loose stools. The Food and Drug Administration dropped this warning in 2003 after determining that initial reports of such effects did not hold up in postmarketing studies. Now olestra may be set to take on a new role: as a way to rid the body of toxicants such as dioxin and polychlorinated biphenyls (PCBs).

“It sounds like a snake oil pitch,” admits chemist Ronald Jandacek, an adjunct professor at the University of Cincinnati College of Medicine who once worked for olestra developer Procter & Gamble. Jandacek and his colleagues fed mice the radioactively tagged toxicant hexachlorobenzene (HCB) and tracked its levels in the brain and liver during a weight-loss-and-regain diet cycle, which parallels the “yo-yo diet” pattern many Americans follow.

In the first weight loss, HCB increased threefold in the brain, fell with weight regain, and increased with the second weight loss. In the liver, HCB acted differently, increasing with weight regain. When the researchers added olestra, fecal excretion of the toxicant soared 30 times, and its accumulation in the brain fell by half. The study details appear in the February 2005 issue of the *American Journal of Physiology—Gastrointestinal and Liver Physiology*.

Jandacek and colleagues have also completed a preliminary study looking at excretion of HCB in mice during normal food intake and fasting. Olestra appears to enhance the rate of excretion during both, with excretion during the fasting period slightly higher than during the fed period.

“Olestra may be a logical means for biological remediation to remove toxicants,” says Bernard Hennig, a professor of nutrition and food science at the University of Kentucky, adding, “[this work] needs to be confirmed in humans.” Jandacek hopes to eventually feed olestra chips to people living in an area with known organochlorine contamination and monitor toxicant excretion.

In a few case reports, feeding olestra chips to human victims of dioxin poisoning has already been shown to reverse effects. A case report in the June 2005 issue of the *Journal of Nutritional Biochemistry* describes a patient exposed to high levels of Aroclor at work. Under the supervision of researchers at the University of Western Australia, Perth, the patient ate 16 grams of olestra chips daily for two years. His adipose Aroclor levels dropped from 3,200 parts per million to 56, and his physical symptoms disappeared.

The Center for Science in the Public Interest, which opposed the removal of olestra warning labels, is cautious about recommending olestra for toxicant removal. “More power to them if it works as a medicine,” says executive director Michael Jacobsen. He warns, however, that olestra blocks the absorption of cancer-fighting carotenoids such as beta carotene and lycopene, and advises people to replenish these nutrients by eating carotenoid-rich foods like carrots and tomatoes at different times than olestra chips.

